# Molecular Insights into Widespread Pseudouridine RNA Modifications: Implications for Women’s Health and Disease

**DOI:** 10.3390/biology15020142

**Published:** 2026-01-14

**Authors:** Qiwei Yang, Ayman Al-Hendy, Thomas G. Boyer

**Affiliations:** 1 Department of Obstetrics and Gynecology, University of Chicago, Chicago, IL 60637, USA; aalhendy@bsd.uchicago.edu; 2 Department of Medical Sciences, Khalifa University, Abu Dhabi P.O. Box 127788, United Arab Emirates; 3 Department of Obstetrics and Gynecology, Sheikh Shakhbout Medical City, Abu Dhabi P.O. Box 11006, United Arab Emirates; 4 Department of Molecular Medicine, Institute of Biotechnology, University of Texas Health Science Center, San Antonio, TX 78229, USA; boyer@uthscsa.edu

**Keywords:** pseudouridine, epitranscriptomics, RNA modification, pseudouridine synthases, women health, reproductive biology, gynecological diseases, sex-specific regulation, female cancers

## Abstract

Pseudouridine (Ψ), the most abundant RNA modification, plays essential roles in shaping RNA structure, stability, and translational output. Beyond cancer, Ψ is dynamically regulated across numerous physiological and pathological contexts—including immune activation, metabolic disorders, stress responses, and pregnancy-related conditions such as preeclampsia—where elevated Ψ levels reflect intensified RNA turnover and modification activity. These broad functional roles highlight pseudouridylation as a central regulator of cellular homeostasis. Emerging evidence demonstrates that Ψ dysregulation contributes directly to the development and progression of several women’s cancers, including breast, ovarian, endometrial, and cervical malignancies. Elevated Ψ levels in tissues, blood, and urine correlate with tumor burden, metastatic potential, and therapeutic responsiveness. Aberrant activity of Ψ synthases such as PUS1, PUS7, and the H/ACA ribonucleoprotein component dyskerin alters pseudouridylation patterns across multiple RNA substrates, including rRNA, tRNA, mRNA, lncRNAs, snoRNAs, and ncRNAs. These widespread modifications reshape ribosome function, modify transcript stability and translational efficiency, reprogram RNA–protein interactions, and activate oncogenic signaling programs. Advances in high-resolution, site-specific Ψ mapping technologies have further revealed mechanistic links between pseudouridylation and malignant transformation, highlighting how modification of distinct RNA classes contributes to altered cellular identity and tumor progression. Collectively, Ψ and its modifying enzymes represent promising biomarkers and therapeutic targets across women’s cancers, while also serving as sensitive indicators of diverse non-cancer physiological and disease states.

## Simple Summary

Pseudouridine (Ψ) is the most common chemical modification found in RNA and helps RNA molecules fold correctly, remain stable, and function properly in cells. Ψ levels also change in many normal and disease conditions, such as during immune responses, metabolic disorders, stress, and pregnancy complications like preeclampsia. Recent research shows that abnormal regulation of Ψ and the enzymes that produce it can contribute to several cancers affecting women, including breast, ovarian, endometrial, and cervical cancers. Higher Ψ levels in blood, urine, or tumor tissue often reflect larger tumors, metastasis, or how patients respond to treatment. Enzymes such as PUS1, PUS7, and dyskerin can alter RNA modification patterns and influence cancer growth. Because of this, Ψ and its modifying enzymes may serve as useful biomarkers and potential therapeutic targets in women’s cancers, while also indicating changes in various non-cancer conditions.

## 1. Introduction

### 1.1. Overview of RNA Modifications and Epitranscriptomics

RNA modifications represent a critical regulatory layer in gene expression, extending far beyond the four canonical ribonucleotides [1]. To date, more than 170 distinct chemical modifications have been identified across all major classes of RNA, including mRNA, tRNA, rRNA, and noncoding RNAs. These modifications, such as pseudouridine (Ψ), N6-methyladenosine (m^6^A), 5-methylcytidine (m^5^C), and inosine (I), fine-tune RNA structure, stability, localization, and translation [1,2,3,4,5,6,7,8,9]. Collectively, these chemical marks and the enzymes that write, erase, and read them form the basis of epitranscriptomics, a dynamic and reversible regulatory system analogous to epigenetic modifications in DNA and histones.

Epitranscriptomic regulation allows cells to rapidly respond to developmental cues [10,11] and environmental stresses by modulating RNA fate post-transcriptionally [12]. Writer enzymes, such as Ψ synthases and methyltransferases, install specific modifications at defined positions on target RNAs. Eraser proteins remove certain marks, adding reversibility to the process. Reader proteins interpret the chemical signatures to influence downstream RNA behavior, including translation efficiency, splicing decisions, RNA–protein interaction networks, and decay pathways. Importantly, the functional impact of these modifications depends on both the type of chemical mark and its precise location within an RNA molecule.

The emergence of high-resolution sequencing technologies has transformed our understanding of the epitranscriptome, revealing its pervasive contributions to cellular identity, organismal development, and disease pathogenesis [13,14,15,16,17,18]. Dysregulation of RNA modification pathways has been implicated in cancer, neurological disorders, metabolic syndromes, cardiovascular disease, and reproductive dysfunction [4,19,20,21,22,23], highlighting their essential role in maintaining homeostasis. As the field continues to evolve, elucidating how distinct RNA modifications coordinate to shape gene expression landscapes remains a central goal in modern molecular biology.

### 1.2. Discovery and Biological Importance of Ψ

**Figure 1:**
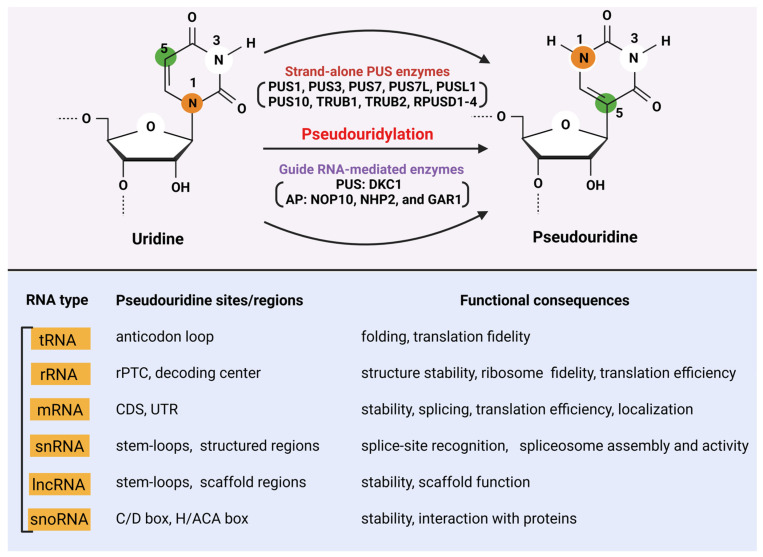
Mechanisms and Functional Effects of Pseudouridine Formation. Schematic overview of how uridine is enzymatically converted to pseudouridine (Ψ), the most abundant RNA modification, and how this process impacts the function of various RNA substrates. Upper panel: In the stand-alone enzyme pathway, a Ψ synthase (PUS) recognizes a specific RNA sequence or structural motif and catalyzes an intramolecular isomerization reaction in which the N1–C1′ glycosidic bond of uridine is cleaved and reformed as a C5–C1′ bond, repositioning the uracil base and generating Ψ without requiring external cofactors or energy. In the H/ACA ribonucleoprotein (RNP)–guided pathway, an H/ACA small nucleolar RNA (snoRNA) or small Cajal body RNA (scaRNA) base-pairs with the target RNA to specify the uridine for modification, while the associated core proteins, DKC1 (the catalytic enzyme), NOP10, NHP2, and GAR1, assemble to form the active RNP complex. DKC1 catalyzes the same isomerization reaction as stand-alone PUS enzymes, yielding Ψ at the guided position. Lower panel: Ψ modifications occur in multiple RNA types, typically at structured or functionally important regions. In rRNA, Ψ is enriched in the ribosomal peptidyl transferase center (rPTC) and decoding sites, where it stabilizes the ribosome and promotes accurate translation. tRNAs carry Ψ in the anticodon loop and conserved stem regions, supporting proper folding and codon recognition, while snRNAs contain Ψ in stem-loops and branch point regions to ensure correct spliceosome assembly. snoRNAs harbor Ψ within stem-loops and other functional domains that maintain snoRNP stability, mRNAs have Ψ in CDS, and UTRs to regulate translation and stability, and lncRNAs feature Ψ in stem-loops and scaffold regions that enhance structural integrity and RNA-protein interactions. The structural consequences of pseudouridylation, including enhanced base stacking, increased local RNA stability, and an additional N1-H hydrogen-bond donor, modulate RNA folding, ribosome function, translation fidelity, and RNA–protein interactions. rPTC: ribosomal peptidyl transferase center; CDs: coding sequence; UTR: untranslated region; AP: associated proteins, snRNA: small nuclear RNA; snoRNA: small nucleolar RNA; lncRNA: long noncoding RNA.

Ψ, often referred to as the “fifth nucleotide”, is the most abundant RNA modification found across all domains of life [24,25]. It was first identified in the 1950s during early investigations of tRNA and rRNA composition, when chromatographic analyses revealed the presence of a unique nucleoside isomer distinct from uridine. Subsequent structural studies uncovered its defining feature, an unusual C5–C1′ glycosidic bond that replaces the standard N1–C1′ linkage of uridine (Figure 1). This structural rearrangement, achieved through an isomerization reaction rather than the addition of a new chemical group, laid the foundation for recognizing Ψ as a central component of post-transcriptional RNA regulation [17,26,27,28].

Biologically, Ψ plays a pivotal role in enhancing the stability, structural integrity, and functional performance of RNA molecules. In tRNA, Ψ contributes to proper folding and accurate codon–anticodon pairing, thereby supporting high-fidelity translation [2,29]. In rRNA, the modification is enriched at functionally critical regions of the ribosome, where it improves base stacking, strengthens hydrogen bonding networks, and stabilizes ribosomal architecture essential for efficient protein synthesis [30]. Beyond structured RNAs, pseudouridylation of mRNA and noncoding RNAs has emerged as a dynamic regulatory mechanism that can influence transcript stability, translation efficiency, and cellular stress responses [4] (Figure 1).

The discovery of Ψ synthases (PUS enzymes), which catalyze the site-specific conversion of uridine to Ψ, revealed a diverse enzyme family with both stand-alone and RNA-guided mechanisms [4,31]. Their evolutionary conservation underscores the fundamental necessity of pseudouridylation across biological systems. More recently, advances in high-throughput sequencing and chemical mapping techniques have uncovered widespread, condition-responsive Ψ deposition in mRNA, linking this modification to developmental processes, immune regulation, and disease states [32,33,34,35]. Together, these discoveries highlight Ψ as a key component of the epitranscriptome, one that integrates structural optimization with regulatory versatility to shape RNA function and cellular homeostasis. As research continues to expand, Ψ is increasingly recognized not merely as a static structural modification, but as a dynamic regulator with broad biological and biomedical implications [36].

### 1.3. Why Women’s Health Is a Unique Lens: Hormonal, Reproductive, and Sex-Specific Pathways

Women’s health offers a distinct and biologically rich lens through which to study human physiology and disease, owing to the intricate interplay of hormonal regulation, reproductive biology, and sex-specific molecular pathways. Unlike many physiological systems that operate similarly across sexes, reproductive and endocrine processes in women are characterized by cyclical, tightly regulated hormonal fluctuations involving estrogen, progesterone, and other ovarian hormones. These hormones modulate a wide array of cellular functions, including gene expression, metabolism, immune activity, and tissue remodeling, providing unique physiological contexts that shape health and disease trajectories [37,38].

The female reproductive system introduces additional layers of biological complexity [39]. The female reproductive system adds further complexity through tightly coordinated processes such as folliculogenesis, ovulation, implantation, pregnancy, and lactation, which require dynamic molecular signaling, RNA regulation, protein synthesis, and immune tolerance [40,41,42]. Reproductive tissues, including the ovary, uterus, and placenta, therefore exhibit specialized molecular environments and heightened sensitivity to disruptions in gene regulatory pathways. Additionally, sex-specific factors such as XX chromosomal composition, X-chromosome inactivation, and stronger immune responsiveness contribute to distinct transcriptional and disease profiles in women [43]. Collectively, these mechanisms highlight the importance of studying women’s health as a unique biological context that offers broader insights into gene regulation and disease pathology.

This review synthesizes current knowledge on RNA pseudouridylation, focusing on the enzymatic functions of Ψ synthases (PUS family members) and their emerging relevance to women’s health and disease. Although Ψ is a highly abundant and conserved RNA modification, its roles in female-specific physiology and disease remain poorly defined. By integrating mechanistic insights from the epitranscriptomic field with growing evidence linking Ψ pathways to female reproductive biology and gynecologic disorders, this review highlights key findings, identifies knowledge gaps, and outlines priorities for future investigation. It aims to provide a conceptual framework for researchers and clinicians seeking to incorporate epitranscriptomic perspectives into women’s health and disease.

## 2. Biology of Pseudouridine

### 2.1. Chemistry and Structure

Chemically, Ψ is an isomer of uridine in which the uracil base is attached to the ribose sugar via a carbon–carbon (C–C) glycosidic bond at position 5 instead of the canonical nitrogen–carbon (N–C) bond at position 1 found in uridine [44]. This subtle structural rearrangement preserves base-pairing capabilities but introduces unique chemical and physical properties that distinguish Ψ from uridine. The C–C linkage in Ψ provides enhanced rotational flexibility of the glycosidic bond, which can influence RNA secondary and tertiary structures. Additionally, Ψ introduces an extra hydrogen-bond donor at the N1 position of uracil, enabling the formation of additional hydrogen bonds. These structural features contribute to increased local RNA stability, enhanced base stacking, and altered conformational dynamics of RNA molecules [45,46].

Functionally, these chemical and structural differences impact RNA folding, stability, and interactions with proteins or ribonucleoprotein complexes. In tRNA, rRNA, and mRNA, pseudouridylation can stabilize critical structural motifs, improve translational fidelity, and modulate ribosome function. Overall, the unique chemistry of Ψ provides a molecular basis for its versatile roles in RNA biology and its importance in regulating gene expression at the post-transcriptional level [24,36].

### 2.2. Biogenesis of Pseudouridine

#### 2.2.1. Pseudouridine Synthases (PUS Enzymes): Context and Function

Ψ synthases (PUS) are a family of enzymes responsible for catalyzing the isomerization of uridine to Ψ in RNA molecules. PUS family comprises a highly conserved group of enzymes that catalyze the site-specific isomerization of uridine to Ψ across diverse RNA species, including tRNA, rRNA, snRNA, and mRNA [17]. In humans, this family contains numerous members, including PUS1, PUS3, PUS7, PUS7L, PUS10, TRUB1, TRUB2, PUSL1, Dyskerin Ψ synthase 1 (DKC1), and RPUSD1–4, that operate either as stand-alone enzymes or as components of RNA-guided H/ACA small nucleolar ribonucleoprotein complexes mediated by DKC1 [4,17]. These enzymes are localized to distinct cellular compartments, including the nucleus, cytoplasm, nucleolus, mitochondria, and Cajal bodies, reflecting the broad functional reach of pseudouridylation. Each PUS member exhibits specific substrate preferences and biological roles. These enzymes generate a dynamic and context-dependent pseudouridylation landscape that modulates RNA structure, stability, translational efficiency, and cellular adaptation to stress, with broad implications for human health and disease, including cancer, congenital syndromes, metabolic dysfunction, and immune disorders [47]. In the context of women’s health, PUS enzyme activity may be modulated by sex hormones, affecting reproductive biology, pregnancy outcomes, and disease susceptibility in a sex-specific manner. Thus, PUS enzymes represent both mechanistic mediators and potential therapeutic targets in female-specific pathophysiology.

#### 2.2.2. Regulation of Pseudouridine Modification Under Physiological Conditions

Ψ is not a static RNA modification; its deposition is tightly regulated in response to cellular and physiological cues. Under normal conditions, pseudouridylation patterns are determined by the activity, expression, and localization of PUS enzymes, as well as by the availability of guide RNAs in guide-dependent mechanisms.

Several physiological factors influence this regulation. PUS activity is highly responsive to the cellular metabolic and stress environment, with factors such as nutrient availability, oxidative stress, and energy status dynamically shaping pseudouridylation patterns across RNA species. Under challenging conditions, including oxidative stress or nutrient deprivation, cells frequently increase pseudouridylation of specific tRNAs and rRNAs to enhance RNA stability, maintain proper folding, and preserve translational fidelity, thereby ensuring continued protein synthesis essential for survival [26,48,49]. These adaptive RNA modifications help stabilize ribosome function and minimize translational errors during stress, while metabolic fluctuations, such as changes in glucose or amino acid supply, further modulate PUS enzyme activity. Through these mechanisms, pseudouridylation serves as a critical link between cellular metabolic state and stress-responsive pathways, enabling cells to dynamically adjust RNA function to maintain homeostasis.

Sex hormones such as estrogen and progesterone exert broad regulatory effects on gene expression not only at the transcriptional level but also through post-transcriptional and epitranscriptomic mechanisms, in part via their receptor, centered regulatory axes that modulate RNA processing, modification, and stability [50]. A study by Zhuang et al. reported that METTL14, the m^6^A methyltransferase, potently binds to the estrogen receptor alpha (ERa) and, therefore, enhances its stability through m^6^A modification [51]. In hormone-responsive tissues—including the endometrium, ovary, breast, and placenta—these signals modulate RNA expression, stability, alternative splicing, and noncoding RNA networks in cyclical or developmental patterns [52], making it biologically plausible that fluctuations in hormonal status also influence PUS activity or its cofactors and thereby shape tissue- and sex-specific pseudouridylation landscapes. Although direct experimental evidence linking estrogen or progesterone to PUS regulation is still limited, multiple RNA-modifying machineries have been implicated in hormone-dependent cancers [53], and estrogen signaling has long been known to modulate post-transcriptional mRNA processing, stability, and ribosome association in hormone-sensitive cells [54]. Given that pseudouridylation affects RNA structure, stability, and translation, hormonal modulation of PUS enzymes could similarly fine-tune Ψ deposition in female tissues across menstrual cycles, pregnancy, and menopause. Such hormone-driven dynamics may influence key biological processes, including proliferation, differentiation, and stress responses, and, when dysregulated, contribute to hormone-dependent cancers, metabolic dysfunction, or reproductive disorders. Understanding how hormonal status intersects with Ψ metabolism represents a promising direction for elucidating sex-specific regulatory biology and disease vulnerability.

Pseudouridylation is highly dynamic and exhibits marked variation across tissues and developmental stages, reflecting its essential role in fine-tuning RNA function during processes such as oogenesis, embryogenesis, stem cell differentiation, and tissue-specific physiology. Emerging evidence demonstrates that Ψ deposition is developmentally regulated; for example, in embryonic stem cells, the Ψ synthase PUS7 modifies specific tRNAs and tRNA-derived fragments (tRFs), which act as modulators of translation initiation and are required for proper germ layer specification during early embryogenesis. Loss of PUS7 disrupts these Ψ-modified tRFs and impairs lineage commitment, highlighting a crucial developmental role for pseudouridylation [55].

Beyond canonical substrates such as rRNA and tRNA, transcriptome-wide profiling has revealed that mRNAs and noncoding RNAs also undergo dynamic and cell-type-specific pseudouridylation [49]. High-resolution mapping studies have shown that Ψ frequently localizes to pre-mRNA regions enriched near splice sites and regulatory motifs, where it can modulate RNA structure, alternative splicing, and 3′ end processing—processes that differ across tissues and physiological contexts [49].

These findings indicate that tissues exhibit distinct “Ψ signatures,” shaped by their unique functional demands and developmental trajectories. During key reproductive and developmental events, such as oocyte maturation, placental development, and embryonic growth, precise regulation of RNA processing and translation likely involves stage-specific patterns of RNA modifications [56]. As cells differentiate and acquire specialized functions, pseudouridylation profiles are reprogrammed accordingly [57]. Collectively, these observations underscore the importance of mapping tissue- and stage-specific Ψ landscapes to understand how RNA modifications govern developmental decisions and maintain tissue-specific homeostasis.

### 2.3. Functional Consequences

#### 2.3.1. Impacts on mRNA Stability, Translation, and Splicing

Pseudouridylation of mRNA can profoundly influence transcript fate by altering RNA stability, translational efficiency, and splicing outcomes [58,59,60]. The isomerization of uridine enhances hydrogen bonding and base-stacking interactions, making the RNA molecule more structurally stable and resistant to nucleolytic degradation [29]. In translation, Ψ-modified codons can improve ribosome decoding fidelity or, in some cases, induce recoding events, including readthrough of premature stop codons [61,62]. Additionally, pseudouridylation within introns or splice sites can modulate pre-mRNA splicing by affecting recognition of splicing motifs or the recruitment of splicing factors, thereby shaping the diversity and abundance of protein isoforms [62,63] (Figure 1).

#### 2.3.2. Effects on rRNA and tRNA Function in Protein Synthesis

In rRNA, Ψ modifications cluster at functionally critical regions of the ribosome, including the peptidyl transferase center and the decoding site. These modifications stabilize rRNA structure, optimize ribosome assembly, and enhance the accuracy and efficiency of protein synthesis. In tRNA, pseudouridylation contributes to proper folding of the tRNA cloverleaf and ensures precise codon–anticodon pairing. Modifications in the anticodon loop and other structural domains support translational fidelity, prevent frameshifting, and maintain efficient protein synthesis under conditions of cellular stress or high metabolic demand [3,64,65,66,67,68] (Figure 1).

#### 2.3.3. Role in RNA–Protein Interactions and Ribosome Remodeling

Pseudouridylation can modulate RNA–protein interactions by introducing subtle structural changes and an additional hydrogen-bond donor, thereby influencing the binding affinity and specificity of RNA-binding proteins [44,69]. This structural contribution has been well documented in biochemical and structural studies showing that Ψ enhances base-stacking, stabilizes local RNA conformation, and strengthens interactions within ribonucleoprotein (RNP) complexes [44,70]. These effects are particularly evident in snRNAs and snoRNAs, where Ψ is enriched within stem-loop or hairpin regions, and other structured domains that serve as key interfaces for spliceosomal and snoRNP protein binding, thereby fine-tuning RNP assembly, architecture and stability [71,72]. In lncRNAs, pseudouridylation within hairpins, scaffold regions, and protein-interaction domains similarly promotes structural integrity and selective recruitment of regulatory RNA-binding proteins, influencing lncRNA-mediated chromatin regulation and post-transcriptional control [73]. In the ribosome, dynamic Ψ modifications, especially within functionally critical rRNA sites, can facilitate conformational rearrangements required for translation initiation, tRNA accommodation, and translocation [30,74,75]. Collectively, by modulating the recruitment or binding efficiency of regulatory RNA-binding proteins, pseudouridylation functions as an epitranscriptomic layer that links RNA modification status with signaling pathways, cellular stress adaptation, and ribosome remodeling under changing metabolic or environmental conditions [26,49] (Figure 1).

## 3. Pseudouridine and Pseudouridylation in Female Cancer

### 3.1. Breast Cancer

Breast cancer remains the most common malignancy among women globally and a leading cause of cancer-related mortality. Its biological heterogeneity reflects extensive genetic, epigenetic, and metabolic alterations. Among emerging contributors, RNA modifications, particularly pseudouridylation, have garnered increasing attention for their roles in regulating gene expression, RNA stability, and cellular behavior. Ψ, the most abundant modified nucleoside, and its synthesizing enzymes (DKC1, PUS1, PUS7) are frequently dysregulated in breast cancer. Elevated Ψ metabolism, either increased circulating/urinary Ψ or overexpression of Ψ synthases, has been linked to tumor growth, metastasis, and poor patient outcomes. Inside tissues and cells, high expression of PUS enzymes leads to increased pseudouridylation of RNA. Pseudouridylation can enhance RNA stability, improve translation accuracy, and support cell growth. In cancer or proliferative tissues, this modification is often upregulated to sustain elevated RNA synthesis and protein production. When Ψ -modified RNAs (e.g., rRNA, tRNA, snRNA) are eventually degraded, Ψ is released. Ψ cannot be recycled, so it accumulates in serum or urine. Thus, high serum and urine Ψ reflects increased total RNA turnover, not decreased RNA stability. Therefore, rapidly growing or malignant tissues exhibit both high pseudouridylation of RNA and high RNA degradation [2,76]. As a byproduct of accelerated RNA turnover, Ψ serves as a measurable biomarker of the hypermetabolic state characteristic of breast tumors.

#### 3.1.1. Liquid Biomarkers: Circulating and Urinary Pseudouridine Levels in Breast Cancer

Early clinical investigations consistently demonstrated that Ψ is elevated in the circulation of breast cancer patients and closely mirrors tumor burden. Serum-based measurements [77,78] showed significantly higher Ψ levels in patients with breast cancer compared with healthy controls, with concentrations increasing progressively with advancing disease stage and the presence of metastatic lesions. Importantly, serum Ψ levels declined in patients who responded to therapy, underscoring its dynamic behavior as a metabolic readout of RNA turnover and tumor activity [79]. These foundational studies established serum Ψ as a clinically relevant, minimally invasive biomarker candidate with clear associations to breast cancer progression, metastatic spread, and therapeutic response.

Across multiple independent cohorts, urinary Ψ has emerged as a robust metabolic indicator of breast cancer that tracks disease severity, reflects accelerated RNA turnover, and offers strong diagnostic and monitoring potential. In a six-nucleoside profiling analysis of 68 patients [80], urinary Ψ showed modest but meaningful associations with tumor size, estrogen-receptor positivity, and treatment modality, contributing significantly to composite nucleoside signatures linked to tumor biology. Its clinical utility is particularly notable in advanced disease: elevated urinary Ψ was detected in 88% of metastatic cases but rarely in localized tumors [81], demonstrating strong discriminatory power for identifying metastatic progression. The micellar electrokinetic chromatography-based profiling further confirmed significantly higher Ψ levels in breast cancer patients versus healthy or benign controls [82], with multivariate models accurately classifying 73% of cases and postoperative declines confirming sensitivity to tumor burden reduction. Comparative analyses with conventional serum markers also highlight its complementary value: urinary Ψ showed clear stage-dependent increases similar to carcinoembryonic antigen (CEA) and was elevated in 36% of patients with distant metastases [80]. Together, these findings establish urinary Ψ as a dynamic, non-invasive metabolic biomarker with diagnostic, staging, and disease-monitoring relevance in breast cancer (Table 1).

Circulating and urinary Ψ levels provide valuable biomarker information but cannot be directly interpreted as reflective of tissue-intrinsic regulation. Establishing robust baseline Ψ landscapes across developmental stages, hormonal cycles, and metabolic homeostasis is therefore critical. Future studies should systematically map tissue-specific Ψ profiles under these conditions and integrate them with circulating and urinary measurements. Such efforts will enable accurate inference of tissue-intrinsic dynamics, clarify the cellular sources of released Ψ, and enhance its translational utility as a biomarker in women’s health and disease contexts.

#### 3.1.2. Molecular Mechanisms: Pseudouridine Synthases in Breast Cancer

**Figure 2:**
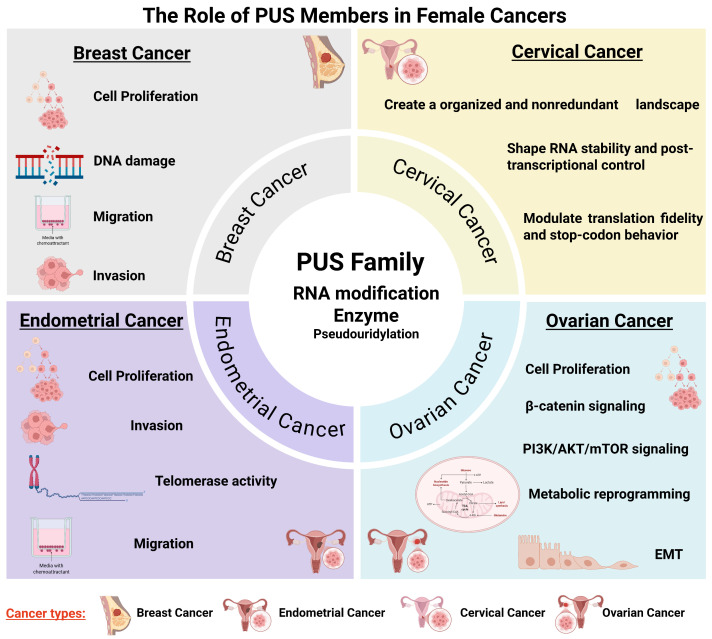
The multifaceted role of PUS members in female cancers distinct cellular and biological pathways. This figure summarizes the PUS-mediated biological functions across major female cancers, including breast cancer, ovarian cancer, cervical cancer, and endometrial cancer. Integrated view highlighting that PUS enzymes function at the intersection of RNA modification, cellular and molecular regulation, underscoring their broad influence on women’s reproductive and overall health.

**Figure 3:**
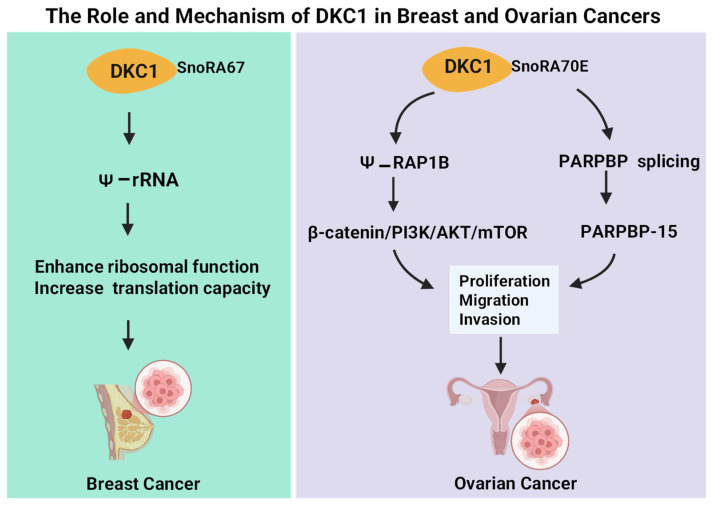
DKC1-mediated pseudouridylation pathways in breast and ovarian cancer. This figure illustrates the distinct mechanisms by which DKC1 contributes to tumor progression in female cancers. Left panel: In breast cancer, DKC1, guided by snoRNA SNORA67, modifies rRNA through pseudouridylation, leading to enhanced ribosomal function and increased translational capacity, which promotes tumor growth [87]. Right panel: In ovarian cancer, DKC1, via SNORA70E, pseudouridylates RAP1B, activating downstream β-catenin/PI3K/AKT/mTOR signaling [94]. In addition, DKC1 regulates PARPBP alternative splicing to generate PARPBP-15. Together, these pathways drive increased cell proliferation, migration, and invasion, contributing to ovarian tumor progression.

DKC1 is a core Ψ synthase that plays a multifaceted oncogenic role in breast cancer by coordinating snoRNA-guided rRNA pseudouridylation, telomerase regulation, ribosome biogenesis, and nucleolar function. Mechanistically, DKC1 catalyzes site-specific Ψ modification of rRNA and stabilizes the telomerase RNA component (TERC), thereby sustaining telomerase activity and promoting cellular immortalization. Elevated DKC1 expression correlates with increased rRNA pseudouridylation, heightened telomerase activity, nucleolar hypertrophy, and poor patient survival [84,85,86]. Large clinical cohorts, including METABRIC, TCGA, and Nottingham, consistently link high DKC1 levels with higher tumor grade and worse prognosis. Mechanistic studies further show that DKC1 overexpression increases snoRNA abundance (e.g., SNORA67), enhances Ψ at key rRNA positions such as 18S-U1445, and elevates translational capacity to support aggressive tumor behavior [87]. Conversely, DKC1 loss or mutation activates a telomere-independent DNA damage response through ATM/p53 signaling, underscoring its essential role in genome stability [88]. Collectively, these findings position DKC1 as a central regulator that integrates pseudouridylation, telomerase maintenance, ribosome output, and genomic integrity to drive the development of high-grade, aggressive breast cancer (Figure 2 and Figure 3).

PUS1 is emerging as a clinically relevant driver of breast cancer, distinct from the ribosome-focused role of DKC1. Responsible for site-specific Ψ deposition in tRNA, rRNA, and other RNAs, PUS1 is consistently overexpressed in breast tumors, with high levels correlating with triple-negative breast cancer, higher tumor grade, and significantly shorter relapse-free and overall survival [89]. Mechanistically, PUS1 promotes proliferation, migration, mitophagy, and PI3K–Akt signaling, while its knockdown reduces TNBC cell growth and invasion. TCGA pan-cancer analyses indicate that PUS1 and other Ψ enzymes are also upregulated in ovarian and endometrial cancers [90]. Loss of PUS1 triggers retrotransposon activation and dsRNA accumulation, activating RIG-I–like receptor pathways and innate antiviral immunity. Pharmacologic suppression with 5-Fluorouracil (5-FU) mimics this effect and enhances anti-PD-1 responses. Thus, PUS1 drives tumor aggressiveness and modulates tumor–immune interactions, highlighting Ψ synthesis as a promising therapeutic and prognostic target in female malignancies.

### 3.2. Ovarian Cancer

Ovarian cancer (Oca) is one of the most lethal gynecologic malignancies, largely due to its asymptomatic onset, late-stage diagnosis, and high rates of chemoresistance. Its complex molecular landscape continues to drive the search for more sensitive biomarkers and actionable therapeutic targets [108,109]. Among emerging regulators of cancer biology, RNA modifications have gained increasing prominence.

Metabolomics analyses have recently highlighted dysregulated Ψ metabolism as a potential early indicator of OCa. A large, nested case–control study [92] analyzing 420 plasma metabolites identified circulating Ψ as one of the strongest predictors of future epithelial OCa risk. Women with higher prediagnostic Ψ levels showed an approximately 2.5-fold increased likelihood of developing OCa, with comparable associations across serous and non-serous subtypes. Although significance diminished after correction for multiple testing, the reproducibility and magnitude of the association suggest that elevated Ψ reflects early disturbances in RNA-modification pathways that precede clinical diagnosis. Complementary metabolic analyses further revealed subtype-specific alterations in triacylglycerols, supporting broader metabolic reprogramming during the earliest phases of OCa development.

Additional support for Ψ as a non-invasive biomarker comes from urinary metabolomics studies. A multi-platform LC–MS investigation [91] combining hydrophilic interaction chromatography (HILIC) and reversed-phase LC effectively distinguishes healthy women, benign ovarian tumors, and Oca, with Ψ emerging as one of the most specific markers of malignant disease, outperforming other cancer-associated metabolites and demonstrating consistent detection across platforms. This specificity reflects its biological origin: Ψ is the most abundant modified nucleoside produced during tRNA turnover, and because it cannot be recycled, it accumulates in circulation and is excreted in urine, making it a sensitive indicator of proliferative activity. These metabolomic data align with transcriptomic and proteomic analyses, demonstrating dysregulation of the RNA pseudouridylation machinery in OCa. Notably, PUS7 is significantly upregulated in tumor tissues across TCGA and GEO datasets [93]., with strong diagnostic performance elevated PUS7 protein expression. Functional enrichment analyses linked PUS7 to RNA processing, cell proliferation, and cellular stress response pathways, suggesting that increased Ψ production and altered Ψ synthase activity contribute to OCa.

At the cellular level, recent studies reveal that Ψ-related regulators—including non-coding RNAs—participate directly in shaping OCa progression. The lncRNA ZFHX2-AS1 [95], which is consistently downregulated in OCa and associated with poor survival, suppresses cell proliferation, migration, and invasion. Mechanistically, ZFHX2-AS1 binds the Ψ synthase DKC1 and attenuates DKC1-mediated pseudouridylation of ARHGAP5 mRNA, reducing its stability and dampening Rho-GTPase–driven Epithelial–mesenchymal transition (EMT) signaling. Restoration of ARHGAP5 reverses this effect, highlighting a functional ZFHX2-AS1/DKC1/ARHGAP5 regulatory axis. In contrast, the H/ACA snoRNA SNORA70E [94] is upregulated and acts as an oncogenic driver by promoting DKC1-dependent pseudouridylation of RAP1B mRNA, stabilizing RAP1B and activating β-catenin and PI3K/AKT/mTOR signaling. SNORA70E also promotes exon-4 skipping in PARPBP, producing a tumor-promoting isoform. Antisense oligonucleotide–mediated inhibition of SNORA70E reduces malignant phenotypes in vitro and in vivo, underscoring its therapeutic relevance (Table 1, Figure 2 and Figure 3).

Across plasma, urine, tissue, and cellular studies, multiple lines of evidence converge on Ψ dysregulation as an early and mechanistically relevant hallmark of OCa. Elevated circulating and urinary Ψ, upregulation of Ψ synthases such as PUS7, and non-coding RNAs that modulate DKC1-dependent pseudouridylation collectively highlight RNA-modification pathways as promising sources of biomarkers and therapeutic targets in OCa.

### 3.3. Endometrial Cancer

EC is the most common gynecologic malignancy in developed countries, and its incidence continues to rise alongside increasing rates of obesity, metabolic syndrome, and hormone-related disorders. Although early-stage EC typically responds well to surgery and adjuvant therapy, advanced or recurrent disease remains challenging to treat, underscoring the urgency of identifying novel molecular drivers and therapeutic vulnerabilities. Recent studies have brought RNA modification pathways, particularly pseudouridylation, into focus as potential contributors to EC pathogenesis.

Pseudouridylation is catalyzed by the H/ACA small nucleolar ribonucleoprotein (snoRNP) complex, with DKC1 as its core catalytic component. In addition to directing rRNA modification, DKC1 stabilizes the telomerase RNA component (TERC) and supports telomerase activity, which is typically elevated in ECs. A recent study has shown that although DKC1 is expressed in normal endometrium, its protein levels are significantly reduced in EC and correlate with poorer clinical outcomes. TCGA data similarly indicate prognostically relevant dysregulation of DKC1, and functional studies suggest a tumor-suppressive role, as DKC1 overexpression reduces EC cell proliferation [96]. In contrast, DKC1-mediated pseudouridylation can function as a potent oncogenic driver in EC. SNORA73B is markedly overexpressed in EC and promotes tumor cell proliferation, migration, invasion, and survival [97]. Mechanistically, SNORA73B increases pseudouridylation-dependent stabilization of target transcripts, including MIB1 mRNA, leading to activating Notch signaling and altering alternative splicing of its host gene RCC1 to generatepro-tumorigenic isoforms. Notably, despite reduced DKC1 expression in EC, SNORA73B, whose function depends on DKC1, remains highly active, revealing a mechanistic paradox that underscores a key knowledge gap in Ψ-dependent regulatory networks and highlights the need for further investigation into pseudouridylation dynamics in EC. (Table 1, Figure 2).

### 3.4. Cervical Cancer

Cervical cancer is a malignant disease arising from the cells of the cervix, the lower portion of the uterus that connects to the vagina. It is most commonly driven by persistent infection with high-risk human papillomavirus (HPV) types, which induce genomic instability and oncogenic transformation [110,111]. Recent transcriptome-wide mapping has revealed that RNA pseudouridylation in cervical cells (HeLa cells) is both extensive and highly regulated. Using bisulfite-induced deletion sequencing, hundreds of Ψ sites have been identified across coding regions and 3′-UTRs, each with quantitative stoichiometry [34]. These studies demonstrate that distinct Ψ synthase (PUS) enzymes install largely non-overlapping sets of Ψ marks. Among the thirteen human PUS members, TRUB1 emerges as the predominant mRNA Ψ writer, responsible for roughly half of all detectable Ψ sites, including highly modified loci in transcripts such as ERH, SCP2, AMFR, CDC6, and FKBP4. Other enzymes, including PUS7, PUS1, PUS3, PUS7L, PUSL1, TRUB2, and DKC1, contribute additional site-specific modifications, establishing a nonredundant division of labor among PUS family members.

The specificity of this system is further supported by distinct sequence motifs that guide PUS targeting. TRUB1 preferentially acts on GUΨCN motifs and poly-U tracts, while PUS7 modifies UVΨAG variants, and TRUB1 favors the GUΨC motif. These stringent sequences and structural preferences underscore the enzyme-encoded rules that shape the HeLa pseudouridylation landscape. Functionally, PUS-dependent Ψ exerts significant control over RNA fate. TRUB1-mediated pseudouridylation stabilizes target transcripts, as TRUB1 depletion lowers Ψ stoichiometry and shortens mRNA half-life, whereas targeted installation of TRUB1-dependent Ψ prolongs transcript stability. In parallel, PUS1 modifies a subset of stop codons, where loss of PUS1 reduces Ψ levels and translational readthrough, while targeted recruitment enhances readthrough efficiency, revealing a regulatory role in translation termination [34].

Together, these findings illustrate a coordinated, multilayered system in which individual PUS enzymes sculpt the Ψ landscape of cervical cancer cells. Through distinct targeting rules and specialized functional outputs, ranging from mRNA stabilization to modulation of translation, PUS-mediated pseudouridylation serves as a central mechanism controlling RNA behavior in cancer-derived cells (Table 1, Figure 2).

### 3.5. Papillary Thyroid Cancer

Thyroid cancer is the most common endocrine malignancy globally, accounting for ~2.1% of all cancer diagnoses and disproportionately affecting women, who represent nearly 77% of cases. Papillary thyroid cancer (PTC) is the most prevalent subtype of differentiated thyroid cancer [112,113]. Although most cases have a favorable prognosis, a subset of women experience aggressive disease characterized by lymph-node involvement, recurrence, or metastasis. Understanding female-specific molecular vulnerabilities is therefore critical for improving diagnosis and targeted therapy in women’s health.

Emerging evidence highlights a role for RNA modifications, particularly Ψ, in shaping PTC biology. Ψ synthases, such as PUS7, introduce Ψ into specific RNA substrates, influencing their stability and function. In PTC, PUS7-mediated Ψ modification of pre-miR-8082 alters microRNA maturation and downstream signaling pathways, ultimately suppressing CD47 and reducing metastatic potential [98]. These findings reveal a mechanistic link between pseudouridylation and thyroid tumor progression, including pathways relevant to the sex-biased incidence of PTC. Given the high prevalence of PTC in women and the physiological sensitivity of RNA-modifying enzymes to hormonal and metabolic environments, Ψ and its regulatory enzymes may represent important female-focused biomarkers and therapeutic targets. Continued exploration of Ψ biology in PTC could therefore advance precision medicine approaches in women’s thyroid health (Table 1).

### 3.6. Cross Talk Between Ψ and Other RNA Modifications in Women’s Cancer

In addition to Ψ RNA modification, other epitranscriptomic mechanisms, particularly RNA methylation, play critical roles in the progression of women’s diseases [114,115,116,117,118,119]. Among these, N6-methyladenosine (m^6^A) is the most abundant and dynamically regulated internal modification in eukaryotic mRNA. m^6^A methylation is installed by a multicomponent “writer” complex centered on the methyltransferases METTL3 and METTL14, together with regulatory cofactors including WTAP, VIRMA, ZC3H13, and RBM15/15B, which collectively determine substrate specificity and subcellular localization. This modification is reversible and can be removed by the demethylases FTO and ALKBH5, allowing dynamic regulation of RNA fate. The functional effects of m^6^A are mediated by “reader” proteins, such as YTH domain–containing factors (YTHDF1–3 and YTHDC1/2) and other RNA-binding proteins, which interpret the modification to regulate mRNA splicing, nuclear export, stability, translation, and decay. Through this coordinated writer–eraser–reader machinery, m^6^A methylation enables rapid, context-dependent modulation of gene expression and contributes to development, stress responses, and disease, including cancer [120].

Increasing evidence indicates that regulators of multiple RNA modifications are not independent but instead exhibit extensive crosstalk, which is closely linked to women’s carcinogenesis, the tumor microenvironment (TME), drug sensitivity, and responses to immunotherapy. In breast cancer, m^6^A modification has been associated with advanced disease stages and poor clinical outcomes, and m^6^A regulatory factors have been validated as important biomarkers that enhance diagnostic accuracy and therapeutic effectiveness [121,122,123]. Comprehensive epitranscriptomic profiling studies further demonstrate that Ψ coexists with m^6^A and other RNA modifications within breast cancer transcriptomes [124]. Similar patterns of epitranscriptomic crosstalk are observed in other hormone-dependent malignancies such as ovarian cancer, where m^6^A RNA modification influences transcripts involved in cell cycle regulation, migration, metastasis, and chemoresistance [125,126,127,128,129], frequently converging with Ψ-regulated shared signaling pathways, including PI3K/AKT [94,130,131]. Collectively, these findings suggest that coordinated regulation by pseudouridylation and m^6^A methylation shapes oncogenic networks in women’s cancers. Integrating detection and functional analyses of both Ψ and m^6^A marks on shared RNA substrates may therefore uncover new epitranscriptomic mechanisms driving female cancer progression and identify opportunities for combinatorial targeting of RNA modification pathways.

## 4. Pseudouridine in Non-Cancer Conditions

Ψ has been increasingly recognized for its role beyond cancer, influencing various non-malignant physiological and pathological processes. Elevated or altered Ψ levels have been associated with adverse pregnancy outcomes, such as preterm birth and preeclampsia, suggesting their potential as a biomarker for maternal and fetal health. In reproductive disorders like polycystic ovary syndrome (PCOS), Ψ may reflect dysregulated RNA metabolism linked to hormonal and metabolic imbalances. Moreover, systemic metabolic conditions, including obesity and related traits, show correlations with urinary and circulating Ψ levels, highlighting their role in energy homeostasis and metabolic regulation. Genetic and metabolic disorders further illustrate the impact of Ψ on RNA stability and cellular function. Interestingly, Ψ is also present in healthy physiological states, reflecting its fundamental role in normal RNA turnover, protein synthesis, and cellular homeostasis. Collectively, these findings underscore the broad relevance of pseudouridylation in women’s health and disease beyond oncology (Table 1).

### 4.1. Preterm Birth

Cervical cerclage is commonly employed to reduce the risk of preterm birth; however, its impact on the vaginal microbiota and associated metabolic profiles remains incompletely understood. Although cervical cerclage does not significantly alter overall vaginal microbiota diversity, recent studies show distinct shifts in microbial composition and metabolite levels during pregnancy [99]. Metabolomic profiling identified 19 altered metabolites, with Ψ among the most notable changes following cerclage. Because Ψ is a stable modification that is not recycled after RNA degradation, higher levels of Ψ in biological fluids typically indicate increased breakdown of Ψ-containing RNAs. In this context, elevated Ψ levels serve as a marker of enhanced RNA turnover rather than RNA stabilization; its altered abundance suggests that cerclage may influence nucleic acid–related metabolic pathways within the vaginal environment. These findings highlight a potential metabolic component to cerclage efficacy, underscoring the need for further research to clarify how Ψ and related pathways contribute to pregnancy outcomes.

### 4.2. Preeclampsia

Preeclampsia (PE) remains a major cause of maternal and perinatal morbidity and mortality, yet its impact on neonatal metabolism is not fully understood. Recently untargeted metabolomic analyses of umbilical cord serum have provided important insights into the metabolic perturbations associated with PE [100]. Comparative profiling of neonates from PE and non-PE pregnancies revealed substantial metabolic alterations, with 159 differential metabolites identified. Notably, Ψ and several other metabolites were significantly elevated in PE neonates. These changes indicate marked disruptions in pyrimidine metabolism, among other pathways, and highlight the metabolic vulnerability of small-for-gestational-age infants born to PE pregnancies.

### 4.3. Polycystic Ovary Syndrome

PCOS is a common endocrine disorder affecting women of reproductive age, characterized by hyperandrogenism, ovulatory dysfunction, and polycystic ovarian morphology [132]. Beyond reproductive abnormalities, PCOS is frequently accompanied by metabolic disturbances—including insulin resistance, obesity, and dyslipidemia—that contribute to long-term health risks such as type 2 diabetes and cardiovascular disease. A study by Yu et al. [101] investigated the clinical impact of acupoint application on in vitro fertilization–embryo transfer (IVF-ET) outcomes in patients with phlegm-dampness type PCOS and explored underlying mechanisms using follicular fluid metabolomics. Metabolomic profiling demonstrated that the treatment group’s follicular fluid more closely resembled that of non-PCOS individuals and differed substantially from untreated PCOS patients, with 34 significantly altered metabolites identified. Notably, acupoint application led to the downregulation of seven metabolites, including Ψ. These findings suggest that acupoint application may improve IVF-ET outcomes in this PCOS subtype, potentially through modulation of metabolic pathways such as pyruvate metabolism, supporting a mechanistic rationale for integrating traditional therapeutic approaches with assisted reproductive technologies.

### 4.4. Pseudouridine as a Metabolic Marker of Fat Distribution

Although obesity research has increasingly incorporated metabolomics to elucidate the biological mechanisms underlying adiposity, comprehensive profiling of metabolites associated with total body fat and its regional distribution across sexes remains limited. Among the metabolites identified in large-scale metabolomic profiling of adiposity traits, Ψ emerged as one of the few molecules specifically associated with fat distribution rather than total fat mass [102]. In a cross-sectional analysis of 3447 individuals across three Swedish cohorts, untargeted LC–MS profiling revealed extensive metabolic perturbations linked to overall adiposity; however, only a small subset of metabolites demonstrated strong specificity for central fat accumulation. Ψ, an established marker of RNA turnover and a product of pyrimidine metabolism, was one such metabolite.

Unlike total fat percentage, Ψ showed a selective relationship with the trunk-to-leg fat ratio, suggesting preferential activation of RNA modification-related pathways in central obesity. This specificity indicates that Ψ may reflect the heightened cellular stress, increased tissue remodeling, or inflammation characteristic of visceral fat depots. Ψ also clustered with metabolites enriched in pathways involving protein synthesis, nucleotide turnover, and sphingolipid metabolism, processes known to be altered in obesity and metabolic syndrome. The study also identified notable sex-specific metabolic differences, although Ψ itself did not show strong sex–interaction effects [102]. Given the adverse cardiometabolic consequences associated with visceral fat accumulation, Ψ may represent a mechanistic link between altered nucleotide metabolism and obesity-related disease pathways, warranting further investigation into its functional role beyond serving as a biomarker.

### 4.5. Baseline Urinary Pseudouridine Levels in Healthy Women: Stability Across the Menstrual Cycle and Implications for Biomarker Studies

Studies assessing urinary modified nucleosides in healthy young women provide essential baseline context for interpreting disease-related alterations. Representative chromatograms of Ψ demonstrate clear and reproducible detection across analytical platforms. Notably, urinary Ψ levels remain highly stable throughout the menstrual cycle, indicating that normal hormonal fluctuations exert minimal influence on its excretion, reinforcing Ψ as a reliable indicator of baseline RNA turnover [105]. Together, these findings highlight the importance of defining robust physiological Ψ levels in healthy populations to support accurate interpretation in clinical and pathological settings. Its consistent physiological profile underscores its potential as a sensitive biomarker, where deviations from baseline may reflect changes in cellular activity, RNA turnover, or emerging disease processes.

## 5. Therapeutic Targeting of Pseudouridylation Machinery

Targeting RNA modifications showed great potential [133]. Telomerase enables the unlimited replicative potential of cancer cells, and its proper assembly depends on the catalytic subunit hTERT, the RNA template hTR, and the RNA-binding protein DKC1. Because DKC1 stabilizes hTR and is essential for telomerase function, it has emerged as a promising therapeutic target in telomerase-dependent cancers. Disrupting the interaction between DKC1 and hTR offers a novel strategy to inhibit telomerase at the assembly stage rather than targeting its catalytic activity. To pursue this approach, accurate structural models of human DKC1 were generated through homology and ab initio modeling, revealing two hydrophobic pockets within the PUA RNA-binding domain suitable for small-molecule docking [106]. Using these models, a docking-based virtual screen of 450,000 compounds identified candidates with high predicted affinity, and several showed inhibitory effects in MDA-MB-231 breast cancer cells. These findings provide proof-of-concept that targeting DKC1 can impair telomerase activity, supporting the development of telomerase assembly inhibitors as a complementary strategy in cancer therapy.

Paclitaxel (PTX) is a standard therapy for triple-negative breast cancer (TNBC), but its use is limited by dose-related toxicity. DKC1 is overexpressed in TNBC and linked to poor outcomes, making it an appealing target to enhance chemotherapy response. The study by Vilarullo et al. examined whether combining PTX with R1D2-10, a DKC1 inhibitor developed by their group, could improve efficacy while lowering PTX requirements [107]. Using human breast cancer cell lines, they assessed cytotoxicity, drug synergy, clonogenic survival, cell cycle effects, apoptosis, and stress markers. The PTX–R1D2-10 combination showed strong synergy, achieving a dose reduction index above 3 and markedly decreasing colony formation. It increased G2/M and sub-G1 populations and enhanced apoptosis without inducing replication stress or DNA damage, suggesting that synergy arises through cell cycle arrest and apoptotic pathways. These results indicate that DKC1 inhibition may enhance PTX sensitivity and reduce toxicity in TNBC, supporting further evaluation in vivo.

Most existing work centers on DKC1, highlighting a substantial gap in understanding how other RNA-modification–targeted therapeutics might contribute to female cancer treatment. Several Ψ synthase (PUS) inhibitors, such as PUS1 inhibitors (e.g., 5-FU, which indirectly suppresses PUS1-driven programs in liver cancer) [90], PUS7 inhibitors (including enhanced-affinity small molecules identified in glioblastoma and neuroblastoma models) [48,134]. However, these agents have not yet been applied or evaluated in female-specific disease models, leaving open the question of whether combined targeting of DKC1 and other RNA-modifying enzymes could yield additive or synergistic therapeutic benefits.

## 6. Limitations and Future Directions

### 6.1. Standardization of Pseudouridine Detection Platforms

Current methods for measuring Ψ, including HPLC, LC–MS/MS, RNA bisulfite–based sequencing, RBS-Seq, and emerging nanopore-based detection, differ substantially in sensitivity, specificity, throughput, and technical complexity. These variabilities limit the ability to compare findings across studies, interpret biological significance, and establish clinically meaningful thresholds. Moreover, pre-analytical variables such as sample collection, RNA integrity, normalization strategies, and data processing pipelines introduce additional layers of inconsistency. To facilitate clinical translation, the field urgently requires standardized analytical platforms, reference materials, and harmonized protocols for Ψ quantification. Establishing consensus guidelines will not only improve reproducibility but also support the development of validated biomarkers and regulatory-grade assays suitable for liquid biopsy applications and large-scale clinical trials.

### 6.2. Integrating Ψ Metrics with Existing Clinical Biomarkers

While urinary, serum, or tissue levels of Ψ have emerged as promising indicators of RNA turnover and cellular activity, their independent diagnostic and prognostic value remains incompletely defined. To realize their full clinical potential, future studies should investigate how Ψ measurements can be integrated with established clinical biomarkers—such as metabolic markers, inflammatory cytokines, or tumor-specific proteins—to enhance disease prediction, monitor therapeutic responses, and refine patient stratification. High-resolution mapping of rRNA pseudouridylation in breast cancer using RBS-Seq [66] has revealed tumor-specific hyperpseudouridylation patterns and substantial interpatient variability in Ψ sites. These detailed modification profiles allow for the stratification of patient clusters and correlate with specific clinical and pathological features, underscoring ribosome modification heterogeneity as a contributor to tumor biology. Importantly, selected tumor-specific Ψ sites identified through RBS-Seq can be validated in minimally invasive liquid samples such as serum or urine, bridging the gap between mechanistic insights and clinical application. This approach provides a translational framework for developing Ψ-based liquid biomarkers that complement conventional clinical assays, offering a novel avenue for dynamic patient monitoring, early detection of recurrence, and personalized therapeutic guidance.

### 6.3. Understanding Tissue-Specific and Context-Dependent Ψ Functions

Most existing studies of Ψ have focused primarily on its circulating or urinary levels, although, while useful as systemic biomarkers, offer limited insight into tissue-specific regulation, distribution, and function. The dynamics of Ψ modification within individual organs or specific cell types remain largely unexplored, leaving key questions about its localized roles unanswered. Understanding how pseudouridylation patterns vary across different tissues under normal physiological conditions, such as during development, hormonal cycles, or metabolic homeostasis, is essential to interpret baseline biological variability. Equally important is characterizing how these patterns are altered in pathological states, including obesity, diabetes, cancer, and endocrine disorders, where dysregulated RNA modification may contribute to disease progression or tissue-specific vulnerability. Integrating tissue-level mapping with systemic measurements will therefore be critical for linking Ψ biology to cellular function, identifying organ-specific signatures, and developing precise diagnostic or prognostic applications that reflect both local and systemic disease processes.

### 6.4. Cellular Sources of Released Ψ

Elevated Ψ levels in cancer patients may originate from both tumor-intrinsic and host-derived sources. On the one hand, increased RNA turnover and dysregulated pseudouridylation within tumor cells contribute substantially to Ψ release, as malignant cells exhibit accelerated RNA synthesis and degradation. Consistent with this notion, increased urinary and serum Ψ levels have been reported in cancer patients and are thought to reflect enhanced whole-body RNA turnover, which is often driven by high proliferative activity in tumors [135]. Moreover, elevated Ψ levels have been correlated with tumor burden and disease progression, and transcriptomic profiling has revealed distinct pseudouridylation patterns in tumor tissues compared with adjacent normal tissues, directly linking altered Ψ dynamics to tumor cell biology [4]. On the other hand, elevated Ψ detected in body fluids such as serum, urine, and saliva may not derive exclusively from tumor cells. Host tissues may also contribute through systemic metabolic adaptations, immune activation, or inflammatory responses associated with cancer. Early studies have suggested that abnormal nucleoside excretion reflects tumor–host metabolic interactions and increased whole-body RNA turnover rather than a purely tumor-specific process [136]. The observation of elevated Ψ levels across multiple cancer types and biological compartments supports the idea that cancer-associated changes in RNA metabolism extend beyond localized tumor cell effects and involve broader systemic responses.

Despite accumulating evidence linking elevated Ψ levels to malignancy, the precise cellular origins of circulating or urinary Ψ remain poorly defined. Most existing studies rely on bulk measurements of Ψ in body fluids, which do not distinguish between tumor-derived and host-derived contributions. Additionally, correlations between Ψ levels and tumor burden or progression do not establish causality or cellular specificity. The lack of spatially resolved or cell-type–specific pseudouridylation analyses limits our ability to determine whether increased Ψ primarily reflects intrinsic tumor RNA turnover, altered pseudouridylation machinery, or systemic host responses such as inflammation and metabolic remodeling. Therefore, future studies should aim to delineate the cellular sources of released Ψ with greater precision. Integrating tissue-specific pseudouridylation profiling, single-cell or spatial transcriptomic approaches, and isotope-tracing strategies may help distinguish tumor-derived Ψ from host contributions. Longitudinal analyses comparing Ψ levels before and after tumor resection or treatment could further clarify the relative roles of tumor burden versus systemic responses. In addition, mechanistic studies investigating how immune activation, metabolic stress, and non-malignant stromal tissues influence RNA turnover and Ψ release will be essential to fully understand the biological significance of elevated Ψ in cancer and to assess its utility as a disease-specific biomarker.

### 6.5. Research Gaps in Non-Malignant Female Diseases

Despite growing interest in Ψ as a biomarker and regulatory RNA modification, its role in non-malignant female diseases remains strikingly underexplored. Most current studies center on cancer, leaving major gaps in our understanding of Ψ dynamics across common yet understudied conditions such as polycystic ovary syndrome (PCOS), endometriosis, uterine fibroids, premature ovarian insufficiency, menstrual cycle disorders, pregnancy complications beyond preeclampsia, and menopausal metabolic changes. These conditions involve profound hormonal, inflammatory, metabolic, and immune alterations, factors known to influence RNA turnover and modification, but systematic analyses of pseudouridylation patterns in these contexts are largely absent. Moreover, virtually nothing is known about how pseudouridylation of specific RNA substrates, including mRNA, tRNA, rRNA, snoRNA, and long noncoding RNAs, might contribute to the pathophysiology of these diseases or serve as diagnostic or prognostic indicators. Expanding Ψ research beyond oncology to encompass the broader spectrum of female reproductive and endocrine disorders will be essential for identifying disease-specific RNA modification signatures, clarifying the physiological functions of Ψ in women’s health, and unlocking new opportunities for biomarker discovery and therapeutic development.

### 6.6. Expanding Pseudouridine Research in Female Cancers

Although recent studies have begun to uncover the role of Ψ in tumor biology, research focused specifically on female cancers, such as breast, ovarian, cervical, and endometrial cancer, remains in its early stages. These malignancies exhibit distinct hormonal regulation, metabolic environments, and immune landscapes that likely shape pseudouridylation patterns in both rRNA and tRNA, as well as the activity of PUS family enzymes and DKC1. Preliminary evidence suggests that aberrant Ψ deposition may influence ribosome function, translational control, and oncogenic signaling pathways in a cancer- and tissue-specific manner. However, comprehensive mapping of pseudouridylation, functional characterization of site-specific changes, and mechanistic studies linking Ψ biology to female tumor progression are still lacking. Expanding research in this area will be essential for identifying female cancer–specific Ψ signatures, clarifying their contribution to tumor heterogeneity and treatment response, and advancing Ψ-based biomarkers or therapeutic strategies tailored to women’s cancers.

### 6.7. Therapeutic Safety of Targeting Essential RNA-Modifying Enzymes

Emerging evidence identifies Ψ synthases as promising therapeutic targets due to their roles in cancer progression, immune modulation, and oncogenic signaling. However, because these enzymes are essential for normal RNA processing and cellular homeostasis, targeting pseudouridylation carries risks of impaired protein synthesis and tissue toxicity. To advance these strategies safely, detailed preclinical studies are needed to define dose–response relationships, tissue-specific effects, and off-target consequences. Selective or transient inhibition of tumor-associated Ψ synthases, as well as combination approaches with immunotherapy or chemotherapy, may help maximize antitumor efficiency while minimizing systemic toxicity.

Collectively, addressing these safety and specificity concerns will be critical for translating mechanistic insights into clinical applications and for developing Ψ-targeted therapies as precise modulators of RNA biology while preserving normal cellular homeostasis.

## 7. Conclusions

Ψ and its modifying machinery, including PUS family synthases, associated proteins, and small nucleolar RNAs (snoRNAs), play essential roles in maintaining RNA stability, translational fidelity, and cellular homeostasis. Dysregulation of pseudouridylation has been implicated in numerous conditions relevant to women’s health, including breast, ovarian, cervical, and EC, metabolic disorders, and pregnancy-related complications, among others. Elevated or aberrant Ψ levels serve as robust biomarkers, reflecting tumor burden, disease progression, metastatic potential, and metabolic or hormonal status. Targeting dysregulated pseudouridylation through inhibitors of enzymes such as PUS1, PUS7, and DKC1 offers a promising therapeutic approach, capable of suppressing tumor growth, modulating immune responses, and enhancing treatments such as immunotherapy, although safety and specificity must be carefully considered.

Advances in single-cell and spatial transcriptomics now allow high-resolution mapping of Ψ modifications across individual cells and tissue microenvironments, capturing heterogeneity and context-specific dynamics in female tissues such as the ovary, uterus, and placenta. These approaches reveal functional microdomains influenced by hormones, reproductive cycles, or disease, providing insights into mechanisms of tumor progression, placental dysfunction, and metabolic stress. Integrating Ψ profiling with clinical, hormonal, and multi-omics data enables personalized risk assessment, patient stratification, and the identification of novel biomarkers or therapeutic targets. Together, these developments highlight the potential of Ψ-based approaches to advance precision medicine, offering new avenues for diagnosis, prognosis, and tailored interventions in women’s health.

## Figures and Tables

**Table 1:** Key Parameters for Characterizing Pseudouridine-Related Biomarkers and Mechanistic Studies in Basic and Translational Research.

Clinical Condition	Target	Sample Types	Clinical Cohort (n/Dataset)	Mechanism	Approaches	Biological Effects	Time	Ref.
Breast Cancer	Ψ levels	Serum	NA	Tumor-driven RNA turnover and metabolism	Radioimmunoassay	Increased Ψ levels in breast cancer patients	1975	[77]
Breast Cancer	Ψ levels	Urine	72	Tumor-driven RNA turnover and metabolism	Biochemical assay	Increased Ψ levels linked to larger tumors and bone metastasis, correlated with ER but not PR	1996	[78]
Breast Cancer	Ψ levels	Serum	NA	Tumor-driven RNA turnover and metabolism	Ψ quantification	Increased Ψ levels correlate with disease progression and patients’ responses to therapy	1983	[79]
Breast Cancer	Ψ levels	Urine	104	Tumor-driven RNA turnover and metabolism	HPLC	Elevated Ψ levels became more frequent and higher with advancing clinical stage	1987	[80]
Breast Cancer	Ψ levels	Urine	22	Tumor-driven RNA turnover and metabolism	HPLC	Urinary Ψ is elevated in metastatic breast carcinoma	1987	[81]
Breast Cancer	Ψ levels	Urine	26	Increased metabolic activity, RNA turnover	Micellar Electrokinetic Chromatography	Urinary nucleosides, including Ψ levels, behave as tumor-associated metabolic markers	2005	[82]
Breast Cancer	Ψ levels	Serum	KORA, Twins UK	Tumor-driven RNA turnover and metabolism	Genetic variants from GWAS, Mendelian randomization analysis	Ψ levels are associated with highest protective value for ovarian and breast cancer	2023	[83]
Breast Cancer	DKC1	Tissue	70	DKC1 regulates telomerase activity	HPLC, cellular assays, telomerase activity	The levels of DKC1 expression and function are associated with tumor progression	2006	[84]
Breast Cancer	DKC1	Tissue	61	DKC1 modulates telomerase activity	Cellular assay, telomerase activity	DKC1 modulates telomerase activity	2008	[85]
Breast & Ovarian Cancer	DKC1	Tissue	1980	High DKC1 expression is closely linked to NP	TCGA, Nottingham cohorts, tissue microarrays, and protein arrays	indicative of adverse clinicopathological features and a worse prognosis	2020	[86]
Breast Cancer	DKC1	Tissue	170	DKC1 enhanced ribosomal activity	tissue DKC1 expression, cellular assay, LC/MS	DKC1 overexpression may drive aggressive tumors and poor prognosis	2020	[87]
Breast Cancer	DKC1	Cell line and mouse model	NA	truncated DKC1 impact growth	Telomerase, P53, and DNA damage	Female heterozygous and male hemizygous truncated *Dkc1* mice showed no detectable phenotype	2008	[88]
Breast Cancer	PUS1	Tissue and cell line	131	Cell proliferation, migration, PI3K-Akt pathway	Tissue array, RNA-seq, cellular assays	PUS1 is a novel biomarker that predicts poor outcomes in BC	2022	[89]
Breast Cancer	PUS family	Tissue	TCGA, Oncomine	Innate antiviral immune signaling	Pan-cancer analysis, cellular and molecular assays, RNA-seq, in vivo	Deregulation of the PUS family, particularly PUS1, may contribute to tumor progression	2025	[90]
Ovarian Cancer	Ψ levels	Urine	22	Metabolic reprogramming	Biochemical assays, integrated analysis	Ψ appears OCa-specific and may serve as a more effective diagnostic marker	2012	[91]
Ovarian Cancer	Ψ levels	Tissue	252	Tumor-driven RNA turnover and metabolism	Nurses’ Health Studies, LC-MS/MS, bioinformatic analysis	Elevated Ψ levels are linked to an increased risk of OCa	2020	[92]
Ovarian Cancer	PUS7	Tissue	TCGA, GEO, Oncomine	Multiple enriched pathways, such as DNA replication	TCGA, GEO, and tissue array	Potential of PUS7 as the diagnostic marker and therapeutic target for OCa	2021	[93]
Ovarian Cancer	DKC1	Tissue	70	Regulate RAP1B via DKC1-mediated Ψ modification	Cellular assays, Ψ detection, RNA-seq, in vivo study	SNORA70E promotes OCa pathogenesis	2022	[94]
Ovarian Cancer	DKC1	Tissue	TCGA, GEO	ZFHX2-AS1 interacts with and attenuates DKC1	Omics data analysis, cellular assays	Novel ZFHX2-AS1/DKC1/ARHGAP5/Rho GTPase axis in OCa progression	2024	[95]
Endometrial Cancer	DKC1	Tissue and cell lines	175	DKC1 may impair telomerase activity	Bioinformatics, expression and cellular assay	Potential for therapeutic approaches targeting DKC1 restoration or modulation	2021	[96]
Endometrial Cancer	SNORA73B	Tissue and cell lines	TCGA	SNORA73B modified the Ψ content in MIB	HPLC, cellular functional assays, RNA-seq, and xenograft model	increased the stability of MIB1 mRNA and protein in EC	2023	[97]
Cervical cancer	PUS members	HeLa	NA	PUS-mediated pseudourylation on mRNA	8 PUS member KD, BID-seq	Identify Ψ sites in rRNA and mRNA	2023	[34]
Papillary thyroid cancer	PUS7	Tissues and cells	TCGA, GEO	PUS7 regulates pre-miR-8082 Ψ	Small RNA ψ modification microarray, MeRIP-PCR, cellular assays	PUS7 suppresses CD47 and PTC cell metastasis	2024	[98]
Preterm birth	Ψ levels	Vaginal	84	RNA turnover	Cervical cerclage, 16S rRNA-seq, metabolomics analysis	Ψ levels were significantly changed during pregnancy after cervical cerclage	2025	[99]
Preeclampsia	Ψ levels	Cord blood samples	29	RNA turnover	LC-MS/MS	Ψ was among the most elevated cord-blood metabolites in preeclampsia neonates	2022	[100]
PCOS	Ψ levels	Follicular fluid	90	RNA turnover	UHPLC-QTOF MS, pathway analysis	Intervention of acupoint application decreased the Ψ levels	2022	[101]
Obesity-related traits	Ψ levels	Plasma or serum samples	3447	Amino acid utilization, lipid signaling, and RNA turnover	Fat mass measurement, LC–MS metabolomics	Ψ levels correlated with fat distribution but were not sex-dependent	2023	[102]
X-linked ichthyosis	Ψ-5’-phosphatase	B-cell lymphoblast and erythrocytes	NA	Converts Ψ-5’-phosphate → Ψ	Biochemistry and cellular and metal-dependent assays	DHD1 functions as the Ψ-5′-phosphatase, showing genetic and sex-related variation	2010	[103]
Healthy subjects	Ψ levels	Urine samples from 77 healthy subjects	77	RNA turnover	Urinary metabolites	Highest excretion values, no significant sex differences in excretion rations among adults	1990	[104]
Normal menstrual cycle	Ψ levels	Urine from women between 17 and 24 ys	NA	RNA turnover	Gas–liquid chromatography	The excretion levels were essentially unaffected by the menstrual cycle for Ψ levels	1977	[105]
Breast Cancer	DKC1	TNBC	NA	Inhibitory effect for DKC1	Library with 450,000 compounds were screened, telomerase activity	Three compounds showed an inhibitory effect	2018	[106]
Breast cancer	DKC1	TNBC	NA	Cell cycle, apoptosis	Cellular assay, DKC1 inhibitor	synergistic effects of DKC1 inhibition and paclitaxel	2025	[107]
Breast cancer	Ψ sites at rRNA	Tissue	34	Intrinsic ribosomal modifications	RBS-seq	Site-specific Ψ mapping reveals ribosomal modifications driving BC pathogenesis	2023	[66]

## Data Availability

No new data were created or analyzed in this study.

## Abbreviations

The following abbreviations are used in this manuscript:

ARHGAP5	Rho GTPase–activating protein 5
β-catenin	Beta-catenin signaling protein
CEA	Carcinoembryonic antigen
CD47	Cluster of Differentiation 47
DKC1	Dyskerin pseudouridine synthase 1
dsRNA	Double-stranded RNA
EC	Endometrial cancer
EMT	Epithelial–mesenchymal transition
ES/ESC	Embryonic stem cells
GEO	Gene Expression Omnibus
H/ACA snoRNP	H/ACA box small nucleolar ribonucleoprotein
hTERT/TERC(hTR)	Human telomerase reverse transcriptase/Telomerase RNA component
HILIC	Hydrophilic interaction chromatography
HPLC	High-Performance Liquid Chromatography
IVF-ET	In vitro fertilization–embryo transfer
LC–MS/LC–MS/MS	Liquid chromatography–mass spectrometry/tandem mass spectrometry
m^5^C	5-methylcytidine
m^6^A	N6-methyladenosine
ncRNA	Noncoding RNA
OCa	Ovarian cancer
PCOS	Polycystic ovary syndrome
PE	Preeclampsia
PI3K–Akt	Phosphoinositide 3-kinase–Akt signaling pathway
PTC	Papillary thyroid cancer (context-dependent)
PTX	Paclitaxel
PUS/Ψ-synthase	Pseudouridine synthase
Ψ	Pseudouridine
RBP	RNA-binding protein
RBS-seq/RBS-Seq	RNA bisulfite sequencing
RCC1	Regulator of chromosome condensation 1
RIG-I	Retinoic acid–inducible gene I
RNP	Ribonucleoprotein
rPTC	Ribosomal peptidyl transferase center
rRNA	Ribosomal RNA
snRNA	Small nuclear RNA
snoRNA	Small nucleolar RNA
TCGA	The Cancer Genome Atlas
TNBC	Triple-negative breast cancer
tRF	tRNA-derived fragment
tRNA	Transfer RNA
TRUB1/TRUB2	TruB pseudouridine synthases 1 and 2
RPUSD1–4	RNA pseudouridine synthase domain-containing proteins 1–4
